# Morphological description and multilocus genotyping of *Onchocerca* spp. in red deer (*Cervus elaphus*) in Switzerland^☆^

**DOI:** 10.1016/j.ijppaw.2022.10.004

**Published:** 2022-11-03

**Authors:** Ralph Manzanell, Anna-Sophia Stocker, Peter Deplazes, Alexander Mathis

**Affiliations:** a Unaffiliated, Bonaduz, Switzerland; b Institute of Parasitology, Vetsuisse and Medical Faculty, University of Zürich, Zürich, Switzerland

**Keywords:** *Onchocerca flexuosa*, *Onchocerca jakutensis*, *Onchocerca skrjabini*, Morphology, Genotyping, Switzerland

## Abstract

Onchocercosis is a parasitic disease caused by over 30 *Onchocerca* spp. (Nematoda: Filarioidea) and predominantly affecting ungulates. Four *Onchocerca* spp. have been described in the European red deer (*Cervus elaphus*). *Onchocerca flexuosa* and *Onchocerca jakutensis* form subcutaneous nodules in the back region. The other two species, *Onchocerca skrjabini* and the lesser-known *Onchocerca garmsi*, are found freely in the subcutaneous tissue of carpal and tarsal joints, and the sternal region, respectively. The presence of *Onchocerca* spp. in eight red deer shot in the hunting season during September 2020 in the Grisons region, Switzerland, was investigated by analysing nodules and free worms in the subcutaneous tissue. The obtained worms were morphologically and genetically identified as *O. jakutensis*, *O. flexuosa* and *O. skrjabini*. The latter two are first reports from Switzerland, and morphological redescriptions of these two species are presented. *Onchocerca skrjabini* and *O. jakutensis* are newly described from the sternal region of deer. One female of *O. jakutensis* was found free in the subcutaneous tissue of the sternal region, an atypical presentation for this species. Phylogenetic analyses were based on four mitochondrial and one nuclear loci, revealing that *O. jakutensis* belongs to a clade which so far only included non-cervid *Onchocerca* spp. Analysis of sequences from this study and GenBank entries revealed two distinct subpopulations of *O. skrjabini*: one from European red deer and another from Japanese serow and sika deer. Morphological identification can be challenging, also because worm location in the host is less strictly determined than previously described. Genetic identification is straightforward for *O. flexuosa*, *O. jakutensis* and *O. skrjabini* for which complete data of five loci are now available whereas genetic data of *O. garmsi* is still lacking.

## Highlights

• First report of *Onchocerca flexuosa* and *O. skrjabini* from red deer in Switzerland.
• Morphological redescriptions are given for *O. flexuosa* and *O. skrjabini*.
• Two subpopulations of *O. skrjabini* from Switzerland and Japan are identified.
• *O. jakutensis* belongs to a different clade than other species with cervid hosts.

## Graphical abstract

**Figure:**
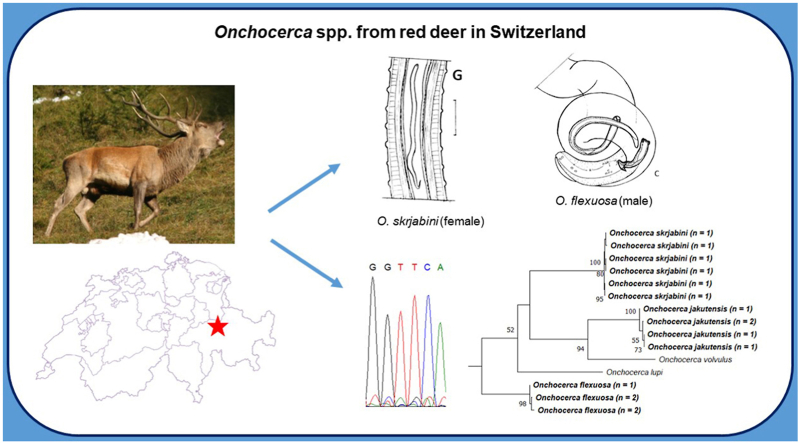

## 1 Introduction

Onchocercosis is a filarial disease caused by over 30 described species of the genus Onchocerca (Filaroidea: Onchocercidae) which are transmitted by insect vectors (Anderson, 2000). *Onchocerca* spp. mainly affect ungulates (Bain et al., 2014; Uni et al., 2015), with two exceptions: *O. lupi* causes ocular infections in carnivores, and *O. volvulus* is the agent of river blindness in humans (Bain et al., 2014) which affects people mainly in tropical Africa and America (World Health Organization, 2022). In addition to *O. volvulus*, humans can suffer from zoonotic infections of different *Onchocerca* species, among others *O. jakutensis* (Koehsler et al., 2007; Wesołowska et al., 2020), which is one of the four species that infect European red deer (*Cervus elaphus*). The adult nematodes of *O. jakutensis* (syn. *O. tubingensis*) are found on the posterior part of the back, the pelvis area and the thighs (Schulz-Key, 1975b; Demiaszkiewicz, 1993; Bosch et al., 2016). Microfilariae are found in the skin overwhelmingly from the abdomen to the sternum and in the base of the ear (Schulz-Key, 1975b). *Onchocerca flexuosa* adults have overlapping predilection sites with *O. jakutensis*, their nodules being found in subcutaneous nodules in the back between the shoulder blades to the lumbar region, in the thigh region and rarely in the front legs (Schulz-Key, 1975b; Plenge-Bönig et al., 1995). Their microfilariae occur in large numbers in the skin of the caudal abdomen and the thighs (Schulz-Key, 1975b). *Onchocerca skrjabini* adults are free in the subcutaneous tissue of the tight skin around the carpal and tarsal joints. Microfilariae are abundant around the site of the adult worms but also on the nose and the base of the ears. The highest concentrations are found in the cutaneous tissue on the ears, especially on the outside (Schulz-Key, 1975b). The fourth species described in European red deer, *O. garmsi*, lives freely in the subcutaneous tissue above the sternum, and its microfilariae are mostly found around the ventral parts of the animal and the base of the ears, very similar to *O. jakutensis* (Schulz-Key et al., 1975).

*Onchocerca flexuosa* and *O. jakutensis* have been described from several European countries (Bosch et al., 2016; Boijsen et al., 2017; Nielsen et al., 2022) and *O. flexuosa* very recently also from Japan (Abd-Ellatieff et al., 2022). *Onchocerca skrjabini* has been described from a few European countries, including Germany (Bain and Schulz-Key, 1974), Finland (Bylund et al., 1981), the Czech Republic (Baruš, 1994), Ukraine (Zhelizniak, 2003) and from Japan (Yagi et al., 1994). *Onchocerca garmsi* has only sparsely been found and is described from Germany (Bain and Schulz-Key, 1976), Poland (Demiaszkiewicz, 1989) and Ukraine (Zhelizniak, 2003).

In Switzerland, so far only adults of *O. jakutensis* have been recorded, with a prevalence of 24% in red deer in the Grisons region (Bosch et al., 2016). All four species have, however, been described in red deer from neighbouring Southern Germany (Schulz-Key et al., 1975; Schulz-Key, 1975b), and, therefore, it might be assumed that they also occur in Switzerland. The aim of this study was to further investigate the presence of *Onchocerca* spp. in Swiss red deer. In addition, two species are morphologically redescribed, and three species are genotyped using five loci. In view of the vast range of distribution of the two species *O. flexuosa* and *O. skrjabini* and the different hosts parasitised in European countries and in Japan, a more detailed morphological (drawings, photographs) and genetic characterisation of individuals was expedient. This is the first comprehensive morphological description of these two species, provided in English.

## 2 Material and methods

### 2.1 Origin of samples, worm preparation and morphological identification

The skins and carcasses of eight wild red deer (*Cervus elaphus*) were selected for investigation by the evident presence of Onchocerca nodules under the skin or in the fascia of the dorsal muscles. They were among the many deer shot during the official hunting season in September 2020 by certified hunters and were processed in the slaughterhouse in Bonaduz (Switzerland). All animals lived in the northern mountains of the Alps, in the catchment area of the river Rhine in the Grisons region, Switzerland.

Worms for further morphological and/or genetic characterisation were collected either from nodules or from the subcutaneous tissue of the skin of the carpal or sternal region. Samples of ca. 8 cm by 10 cm were cut off from the skin removed from deer. The presence of *O. skrjabini* was investigated by adding methylene blue, to stain the parasites, and by using the dissecting microscope (Fig. 1A). Reliable results were obtained after digesting the shaved skin tissue (see below) until only the nematodes remained (Fig. 1B).

**Fig. 1:**
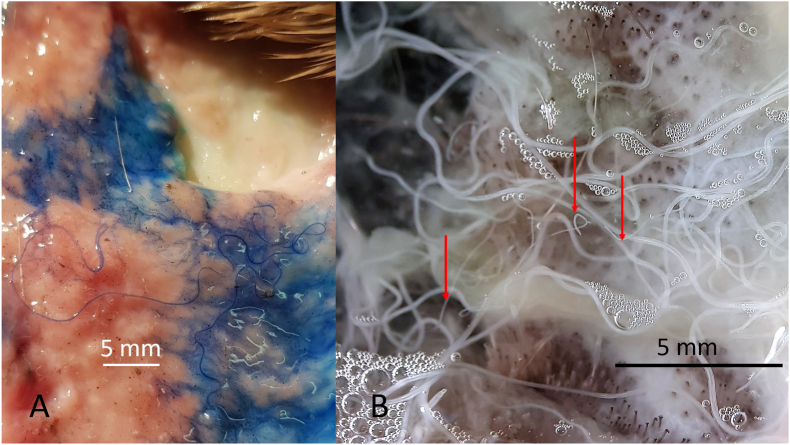
*Onchocerca skrjabini* in situ in carpal skin. **A**. Freshly cut piece of skin shown from inside (note turned around piece of fur on top). Added methylene blue stains the *O. skrjabini* specimens and makes them visible for the naked eye. **B**. Parasitic burden in the same piece of skin in process of digestion. Hair follicles seen as dark dots. Fragments of *O. skrjabini* show different body width. Note very thin anterior head-ends (arrows). (For interpretation of the references to colour in this figure legend, the reader is referred to the Web version of this article.)

Digestion of tissue and isolation of adult worms: The nodules and skin samples were incubated at 58 °C in a subtilisin-enzyme solution (10% v/v) (Enzyrim OSA, Bauer Handels GmbH, Switzerland), buffered at pH 8.0 (109888 Titrisol, Merck, Germany) and 2 drops of Mollescal-C (Bauer Handels GmbH) per 10 ml. Samples were incubated between 2 h (small nodules) to 12 h (thick skin samples). After lysis, specimens were fixed and stored in 70% ethanol and examined with a light microscope with differential interference contrast optics. Drawings were done using an optical Leitz drawing tube mounted on the microscope. The Onchocerca specimens were cleared in chloro-lactophenol if necessary. Identification was done according to the keys of Bain (1981) and other relevant descriptions (Bain and Schulz-Key, 1974; Demiaszkiewicz, 1989). No histopathology analyses were done.

### 2.2 DNA isolation

Additional nodules used for genetic characterisation only were dissected with scissors and forceps. A piece of about 2 cm or several smaller pieces of individual worms were put in 50 μl of PBS (phosphate-buffered saline) and homogenized for 30–60 s using a motor-driven pestle. DNA was then isolated with the Qiamp DNA mini kit (Qiagen, Germany) according to the protocol for DNA purification from tissues (elution volume 200 μl).

### 2.3 PCR and sequencing

PCRs targeting the mitochondrial NADH-dehydrogenase 5 (*nd*5), 12S rDNA, 16S rDNA, cytochrome *c* oxidase subunit 1 (*cox*1), and the nuclear 5S intergenic spacer sequence (5S IGS) were done with the primers and conditions listed in supp. Table 1. Amplifications were carried out in a C1000 touch thermal cycler (BioRad, Switzerland). A total volume of 50 μl per reaction was used, consisting of 19 μl ddH_2_O, 25 μl Multiplex PCR master mix (Qiagen), each 1 μl of the forward and reverse primer (10 μM) and 4 μl of the isolated DNA. The PCR programs are given in supp. Table 1. In cases of weak amplicon production, PCR was repeated with double the amount of DNA or by running up to 50 cycles. Amplicons were analysed on a 1.5% agarose gel stained with GelRed (Biotium, USA) and purified using the Minelute PCR purification kit (Qiagen). The 5S IGS amplicons of about 400 bp were excised from the agarose gels and extracted using the Minelute Gel extraction kit (Qiagen). Amplicon concentrations were measured using the NanoDrop ONEc (Thermo Scientific, USA), and the samples were prepared according to the sequencing company's information for economy run sequencing (Microsynth, Switzerland). Sequences containing ambiguous bases were additionally sequenced with the reverse primer. Sequences were submitted to GenBank under the accession numbers ON854615–ON854658 (Table 1).

**Table 1:** Overview of the Onchocerca specimens used for morphological description, genotyping, and GenBank accession numbers.

Onchocerca species	Specimen						GenBank accession number				
	Isolate	Sex	Red deer	Collected from nodules or free in subcutaneous tissue	Region in deer	Morphological description	*nd*5	12S rDNA	16S rDNA	*cox*1	5S IGS
*O. flexuosa*	OF_A	Female	B	Nodule	Back	No	ON854615^a^	ON854627	ON854638^a^	ON854644	--c
	OF_B	Female	B	Nodule	Back	No	ON854616	ON854628^a^	ON854638^a^	ON854645	ON854655
	OF_C	Female	E	Nodule	Back	No	ON854615	ON854627^a^	ON854638	ON854644^a^	ON854656^a^
	OF_D	Male	G	Nodule	Back	Yes	ON854617	ON854628^a^	--c	ON854645^a^	ON854656
	OF_E	Male	B	Nodule	Back	Yes	ON854615^a^	ON854628^a^	ON854638^a^	ON854646^a^	ON854655^a^
	OF_F	Female	E	Nodule	Back	Yes	ON854615^a^	ON854628	ON854638^a^	ON854646	ON854655^a^
*O. jakutensis*	OJ_A	Female	A	Nodule	Hind leg	No	ON854618	ON854629	ON854639	ON854647	--c
	OJ_B	Female	C	Nodule	Hind leg	No	ON854618^a^	ON854630(29)^b^	ON854639^a^	ON854647^a^	ON854657
	OJ_C	Female	D	Nodule	Pelvis/hind leg	No	ON854619	ON854629^a^	ON854640	ON854648	ON854657^a^
	OJ_D	Female	F	Nodule	Hind leg	No	ON854620	ON854631(29)^b^	ON854639^a^	ON854649	--c
	OJ_E	Female	D	Free	Sternum	No	ON854620^a^	ON854632	ON854639^a^	ON854649^a^	ON854657^a^
*O. skrjabini*	OS_A	Male	D	Free	Sternum	Yes	ON854621	ON854633	--c	ON854650	--c
	OS_B	Female	D	Free	Sternum	No	ON854622	ON854634(33)^b^	ON854641	ON854651	--c
	OS_C	Male	E	Free	Front leg	Yes	ON854623	ON854633^a^	ON854642	ON854654^a^	--c
	OS_D	Female	H	Free	Front leg	No	ON854622^a^	ON854633^a^	ON854643^a^	ON854652	--c
	OS_E	Female	H	Free	Front leg	No	ON854624	ON854635	ON854643	ON854653	--c
	OS_F	Female	E	Free	Front leg	Yes	ON854623^a^	ON854633^a^	ON854643^a^	ON854654	ON854658
	OS_G	Male	H	Free	Front leg	Yes	ON854625	ON854633^a^	--c	ON854650^a^	--c
	OS_H	Female	D	Free	Front leg	No	ON854626	ON854636(33)^b^	ON854643^a^	ON854652^a^	--c
	OS_I	Female	E	Free	Front leg	Yes	ON854625^a^	ON854637(33)^b^	--c	ON854650^a^	--c

a This sequence is identical to that of another isolate and was therefore not separately submitted to GenBank.

b In the alignments, this sequence is identical to the trimmed one of another isolate (indicated in brackets) but the whole sequence was submitted separately to the GenBank.

c No sequence could be obtained.

### 2.4 Data analysis

Mega 11 (Tamura et al., 2021) was used for assessment of the electropherograms, alignments and phylogenetic analyses. A test for Best-Fit Substitution Model was run, and the substitution model with the lowest BIC scores (Bayesian Information Criterion) were selected to generate phylogenetic trees using the Maximum Likelihood mechanism with 500 bootstraps. GenBank accession numbers of additional sequences of filarial species used in this study are presented in supp. Table 2.

**Table 2:** *Onchocerca* spp. infection status of investigated red deer.

Deer	Infection with Onchocerca species		
	*O. jakutensis*	*O. flexuosa*	*O. skrjabini*
A	+ (7)	+ (0)	n.i.
B	+ (0)	+ (23)	neg.
C	+ (5)	neg.	neg.
D	+ (14)	neg. (1)	+ (3)
E	+ (6)	+ (6)	+ (3)
F	+ (11)	neg.	n.i.
G	+ (2)	+ (1)	+ (0)
H	+ (0)	+ (0)	+ (3)

+ = species verified morphologically; number of specimens (total = 85) identified at the *nd*5 genetic locus provided in parentheses; neg. = no infection with this species; n.i. = not investigated.

## 3 Results

A total of 86 Onchocerca specimens were morphologically identified from eight deer.

They belonged to *Onchocerca jakutensis*, a species already known to occur in Switzerland (Bosch et al., 2016), to *O. flexuosa* and *O. skrjabini*, which are both new records for Switzerland. The latter two species are therefore described in detail, both morphologically (see 3.1) and genetically (see 3.2).

Eight Onchocerca individuals were used for detailed morphological description. The same eight individuals and five others were subjected to detailed genetic analysis (Table 2). An additional 72 worms were retrieved from other nodules, putatively also suitable for antigen isolation in the frame of another project. All these worms were analysed at the *nd*5 locus, and a more detailed genetic analysis was done on 7 specimens (Table 2; for features of these worms with regard to sex, etc., see supp. Table 3). *Onchocerca jakutensis* was identified in all eight deer with evident nodules, *O. flexuosa* in 5 out of 8. *Onchocerca skrjabini* was retrieved from the skin of the carpal region of 4 deer and also from the sternal region in 2 of them. Double and triple infections were recorded as shown in Table 2.

**Table 3:** Obtained sequences and their length and identity with sequences of Onchocerca species from GenBank. In parentheses: best matches with species with low identity (indicating lacking corresponding sequences in GenBank).

**Isolates** Locus	**Length** **(bp)**	**Identity** **(%)**	**Species (accession nr.)**
**OF_A – OF_F**			
*nd*5	388–397	99.8–100	*O. flexuosa* (HQ214004, DQ523775, LT732694)
12S rDNA	410–453	99.7–100	*O. flexuosa* (MK391043)
16S rDNA	406–416	100	*O. flexuosa* (LT732686, DQ523759, MZ713089)
*cox*1	615–618	99.5	*O. flexuosa* (AP017692)
5S IGS	159	99.4	*O. flexuosa* (DQ523784)
**OJ_A – OJ_E**			
*nd*5	397	99.8–100	*O. jakutensis* (KU886066, KU886067)
12S rDNA	399–443	99.7-100	*O. jakutensis* (HQ717719, DQ523745, HQ717720)
16S rDNA	408–416	99.7–100	*O. jakutensis* (DQ523758)
*cox*1	616–622	99.3–100	*O. jakutensis* (MK491767, KT001213)
5S IGS	159	93.1	(*O. gutturosa* (DQ523785))
**OS_A – OS_I**			
*nd*5	397	98.6–98.9 92.0–93.1	Unspecified microfilariae (KU886068) (*O. ochengi* (AY462878), *O. lienalis* (AY462892, *O. lupi* (JF758476))
12S rDNA	357–425^a^	97.7–98.0	*O. skrjabini* (AM779804)
16S rDNA	396–409	95.5–95.7	(*O. jakutensis* (DQ523758), *O. fasciata* (DQ523757))
*cox*1	612–620	97.6–98.1	*O. skrjabini* (AM749271)
5S IGS	146	81.8	(*O. flexuosa* (DQ523784))

a sequenced with reverse primer.

Nodules of *O. jakutensis* were found adhering to the skin from the lower back and femoral region embedded in the connective tissue of the subcutis. Most *O. flexuosa* specimens were isolated from nodules within the fascia of dorsal muscles along the back from the scapular region to the sacrum, and a few in the femoral region. The nodules of this species remained on the carcass after the skin was removed, whereas the nodules of *O. jakutensis* adhered to the removed skin. Females were obtained after digestion of the nodules allowing to collect unharmed individuals. Males were found inside or in the outer membranes around the nodules of *O. flexuosa* and *O. jakutensis*.

In contrast to these two species, which live convoluted in nodules, *O. skrjabini* was found outstretched and in large loops in the connective tissue from the hair follicles down to the periost. Only fragments could be collected of the relatively long females, most likely as a consequence of removing the skin from the carcass. Assigning the fragments to individual specimens becomes questionable, particularly if the worm burden is high (Fig. 1B). In addition, fewer fragments with tail/head endings were found amongst the numerous midbody parts. The extremities tend to get lost in the tissue remaining on the host animal. Fig. 2 documents the high worm burden in the sternal skin sample of deer D (Table 2). In this sample, the fragments with taxonomic elements were identified as *O. skrjabini*, but one fragment was identified as *O. jakutensis* using genetic analysis (Table 1, isolate OJ_E).

**Fig. 2:**
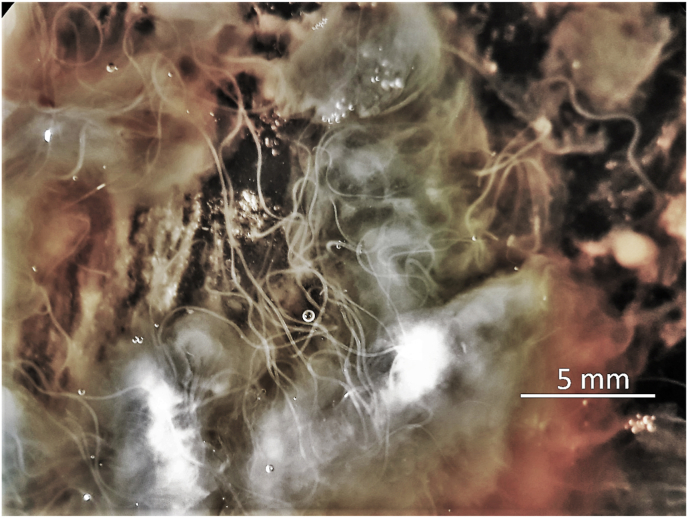
Worm burden in situ of sternal skin in the process of digestion. Most of these worm fragments could be identified as *Onchocerca skrjabini*, no *O. garmsi* was found.

### 3.1 Morphological description

All worms used for morphological description were genetically confirmed and are marked in Table 1. In addition to the specimen used for drawings, the measurements of one more male and female specimen of *O. flexuosa* and two more male and female specimens of *O. skrjabini* are added in parentheses.

#### 3.1.1 Onchocerca flexuosa

Adult worms are white-brownish, opalescent. The intestinal tract of some specimens is brown coloured, in other specimens, all internal organs are brown (Fig. 4, Fig. 9). Mouth opening minute in both sexes, smaller than 2 μm in diameter. Oesophagus clearly divided into anterior muscular and wider and longer posterior glandular part.

##### 3.1.1.1 Male

Body length 85.7 mm (44.7 mm). Oesophagus very long with 8.5 mm (7.65 mm); muscular part of oesophagus 1.2 mm (0.8 mm). Nerve ring at 350 μm (300 μm) from anterior end. Body slowly tapering anteriad from a diameter of 290 μm (230 μm) at the end of oesophagus to 190 μm (180 μm) at nerve ring (Fig. 3A). Cuticle with well-defined, fine transverse annulation, narrowing towards both ends. Posterior part of body ending in narrowing spiral with three tight terminal coils (Fig. 3, Fig. 4). Tail 340 μm (380 μm) long with wide alae. Papillae distributed behind the cloaca as shown in Fig. 3C: One pair medially behind and 4 pairs laterally to the cloaca. An additional group of two pairs situated between cloaca and tail tip and one distal pair close to the tail tip. The phasmids ending in pointed spines at the tail tip. Spicules unequal and dissimilar. Left spicule 840 μm (790 μm), right spicule 230 μm (220 μm). Left spicule with cylindrical proximal shaft and 2.5 times longer distal part where spicule thins to membrane with margins bend ventrad to form tube ending at tip. Right spicule stout, with distal ventral groove and massive hook as dorsal prominence (Fig. 3, Fig. 5).

**Fig. 3:**
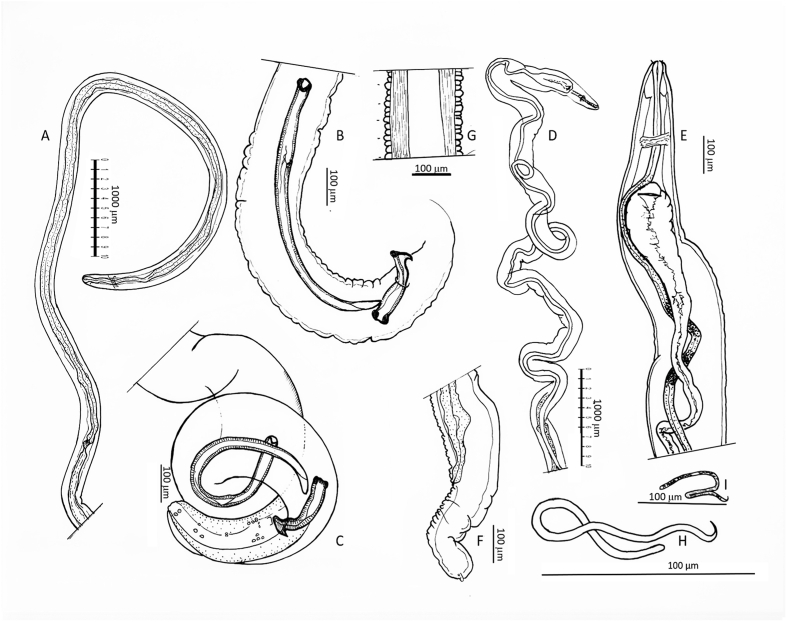
*Onchocerca flexuosa*: **A**. Head end of male. **B**. Spicules in situ with thorn like hook of right spicule protruded and tip of left spicule at proximal entrance of ventral groove of right spicule. **C**. Tail of male showing alae and distribution of papillae. **D**. Head end of female showing vulva with unpaired uterus, oesophagus-intestinal junction depicted and marked with dots at posterior end. Note loose course of uterus with loop. **E**. Head end of female with oesophagus and expansion of ovejector before vulva. **F**. Round tail end of female with spiky phasmid at base of flaps. **G**. Longitudinal section in posterior half of female body showing peculiar repetition of one bigger transversal cuticular ring (marked with dot at left), followed by two smaller rings. **H, I**. Microfilaria, in **I** in same scale as microfilaria in Fig. 10J as comparison.

**Fig. 4:**
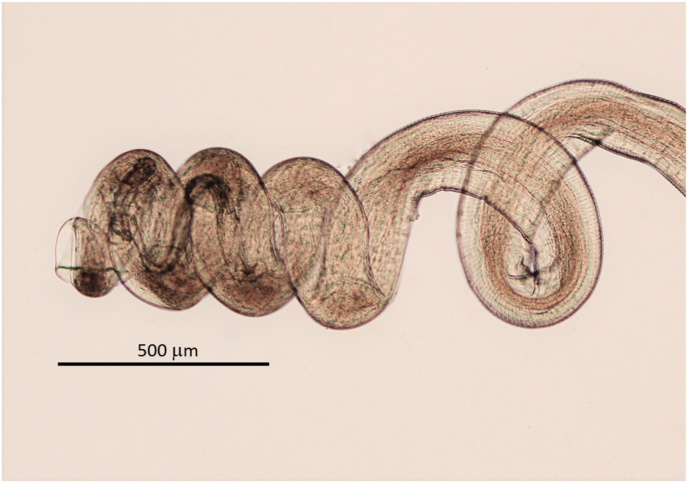
*Onchocerca flexuosa* male tail, typically ending in three tight coils.

**Fig. 5:**
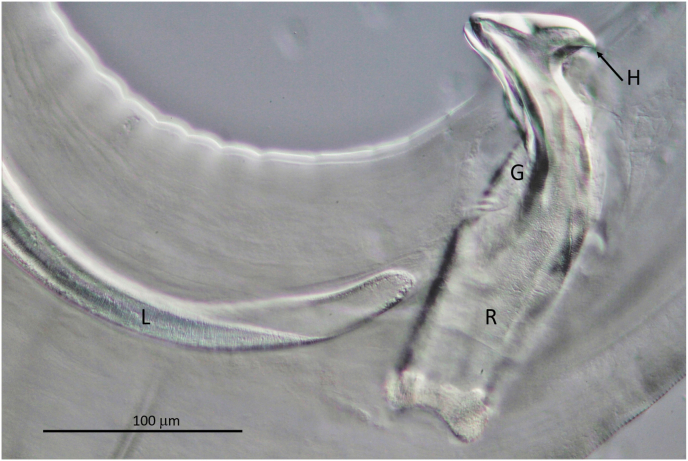
*Onchocerca flexuosa*: Protruding right spicule showing massive hook (H) at dorsal side and groove (G) at ventral side, serving as a gubernaculum to fine tip of left spicule. L: tip of left spicule; R: right spicule.

##### 3.1.1.2 Female

**Fig. 6:**
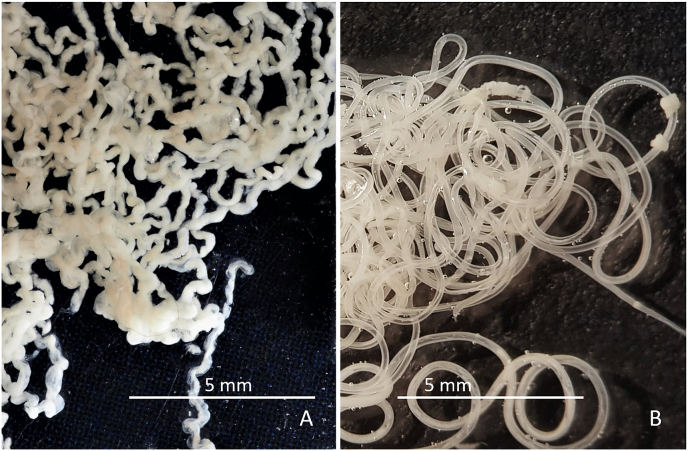
**A**. *Onchocerca flexuosa* female: Typical curly body freed from nodule tissue by digestion. **B**. In comparison a female of *O. jakutensis* after the same digestive treatment.

The enzymatically freed female was found wound up in many loops forming one lump. Body curled (Fig. 6), fragmented in 19 pieces, with total length of 378 mm. Cuticle of females heteromorphous over full length of body, in contrast to cuticles of males.

**Fig. 7:**
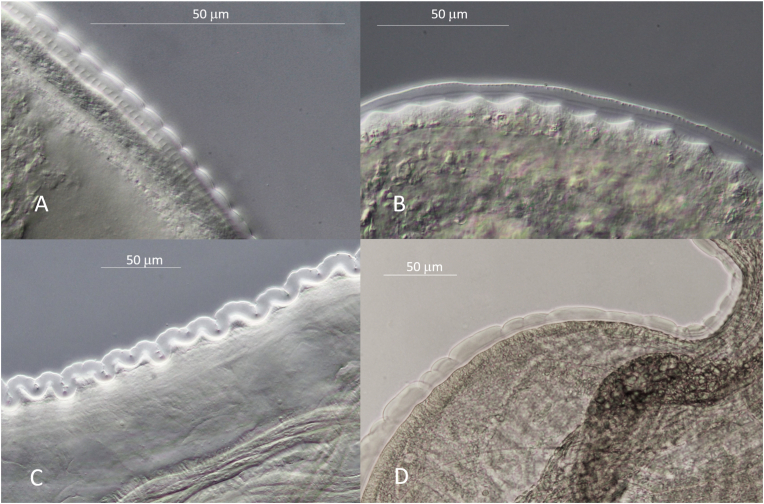
*Onchocerca flexuosa*: Heteromorphous cuticle of female: **A**. Onchocerca-similar cuticle in two layers, with fine striae in medulla, but lacking ridges on surface. **B**. Medullar waves without cuticular ridges. **C**. Entire cuticle forming transversal rings. **D**. Cuticle with repeated pattern of one bigger, followed by two smaller transversal rings.

**Fig. 8:**
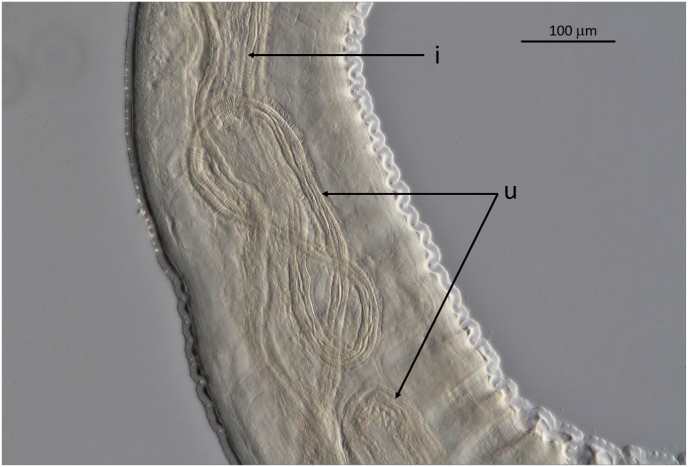
*Onchocerca flexuosa*: Relation between vast internal space in body and thin intestine (i) and unpaired uterus forming loops (u).

**Fig. 9:**
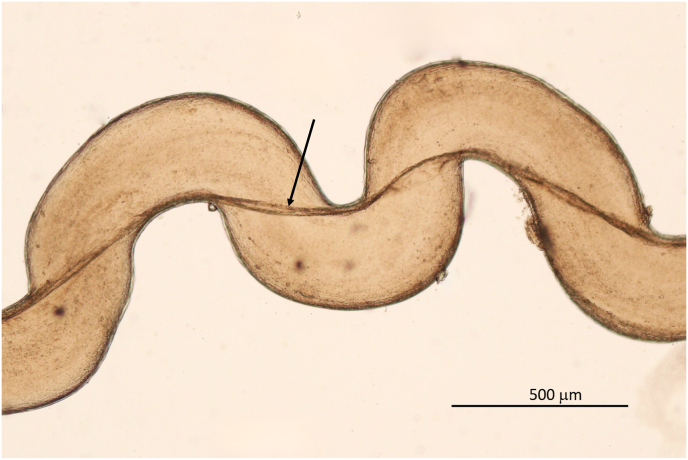
Detail of *Onchocerca flexuosa* female: Uterine tubes (arrow) straightened inside curly body.

Surface of front and back end with fine rounded transversal annulations over a clear, transparent deeper cuticular layer. Surface of cuticle close to cloaca of one female forming Onchocerca-typical ridges with striae in deeper layer with ratio 1 : 4 respectively. Adjacent to midbody ridges of upper cuticle transformed to transversal rounded cushions, with striae underneath still visible (Fig. 7A). At midbody cuticle with rudimentary ridges on smooth surface, but basis of cuticle forming transversal ridges (Fig. 7B), changing over to undulation of entire cuticle (Fig. 7C). In some posterior parts cuticle transformed to transversally repeated pattern of one bigger swelling followed by two smaller swellings (Fig. 3, Fig. 7D). Oesophagus 8.5 mm (6.9 mm) long, with slightly thicker and much longer glandular part. Nerve ring at 220 μm (200 μm), vulva at 370 μm (350 μm) from anterior end. The anterior 2 mm of body tapering slowly, then more rapidly from just behind the vulva to the blunt head end. Width at nerve ring 130 μm (120 μm), at vulva 180 μm (150 μm), at posterior end of oesophagus and midbody 200 μm (220 μm) (Fig. 3D). Ovejector enlarged to more than half of diameter of body (Fig. 3E). Intestine and uteri forming loops inside spacious female body (Fig. 8). Tail 310 μm (550 μm) long, conical, end rounded, with two prominent phasmids and two protuberances at tip (Fig. 3F). In other, degenerating females, the uterine tubes appeared very stretched inside a tightly undulated body, which becomes very fragile (Fig. 9).

##### 3.1.1.3 Microfilaria

Microfilaria (measured inside distal unpaired uterus) unsheathed, very thin, 3–4 μm in diameter, 200 μm (195–200 μm) long (Fig. 3H and I).

#### 3.1.2 Onchocerca skrjabini

Adult worms are whitish, opalescent very thin and difficult to detect by the naked eye in situ (Fig. 1). Mouth opening minute in both sexes, smaller than 2 μm in diameter. Oesophagus of one male and of two female specimens with well distinguished muscular and glandular part. In all other specimens the transition from the muscular to the glandular part was not clearly distinguished, whether by an increase in diameter nor by a sudden onset of glandular cells. The glandular cells only gradually increased in number towards the posterior end.

##### 3.1.2.1 Male

**Fig. 10:**
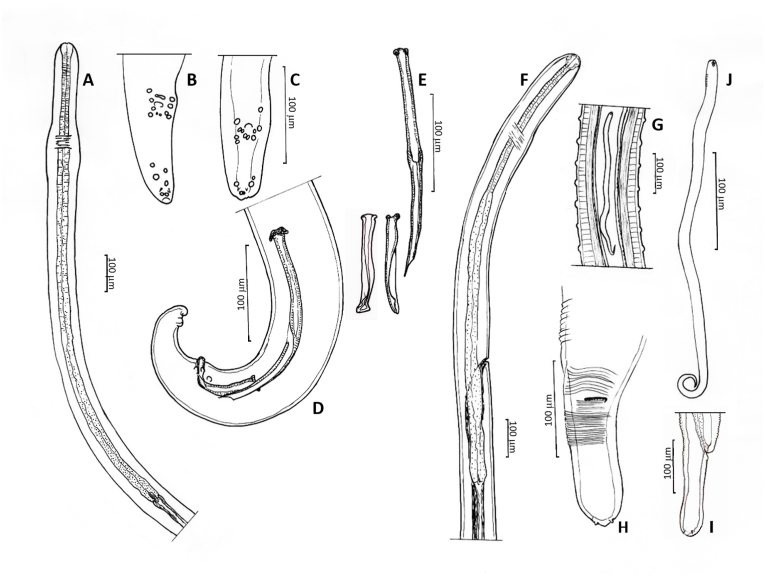
*Onchocerca skrjabini*: **A**. Head end of male, with undifferentiated oesophagus. **B**, **C**. Distribution of papillae on male tail of two different individuals. **D**. Lateral view of male tail with spicules in situ. **E**. Longer left and smaller right spicule. Right spicule in two views to show ventral groove in distal part: on right hand side in lateral view, on left hand side in ventral view, showing dorsolateral knob on tip. **F**. Female head end, with differentiated oesophagus, vulva in distal forth of oesophagus. **G**. Longitudinal section in small anterior part of female, showing unpaired part of uterus filling out anterior body with relatively large microfilaria in it. Cuticular ridges over striae in ratio 1 : 4. **H**. Tail end of female, with two phasmids spaced apart. Fine cuticular transversal annulation is indicated. **I**. Tail end of a second female, lateral view (in smaller scale). Note slight club shape of both. **J**. Microfilaria (intrauterine) with fine striation hinted at the neck, subapical oval marking with ridges.

Body length 35 mm (27 mm, 36 mm), with a diameter at midbody of 75 μm (80 μm, 80 μm), with slight dilatation of body diameter at level of nerve ring. Cuticle with thin transverse annulations, getting finer towards both ends. Oesophagus length 1300 μm (1090 μm, 1150 μm), the 1090 μm long oesophagus with 400 μm muscular part. Nerve ring at 270 μm (250 μm, 260 μm) from anterior (Fig. 10A). Tail 100 μm (85 μm, 100 μm) long, with alae (Fig. 10C). Spicules unequal and dissimilar. Left spicule 228 μm (257 μm, 245 μm), right spicule 95 μm (110 μm, 105 μm) long, both with well-developed heads. Left spicule with distal half flattened with margins bend ventrad to form a closed tube with clearly visible proximal and distal opening. Right spicule forming a ventral groove in distal half, the distal end enlarged dorsally with a subterminal rounded knob (Fig. 10D and E). Papillae in a pericloacal and a distal group. An unpaired precloacal papilla, 4 pairs of papillae laterally to cloaca and one small pair medially behind the cloacal opening. Posterior group with 3 pairs of papillae: One terminal pair close to tip of tail and 2 subterminal pairs. Distribution slightly varying between specimens (Fig. 10B and C). Pair of phasmids opening in a spine between the terminal and subterminal pair of papillae.

##### 3.1.2.2 Female

**Fig. 11:**
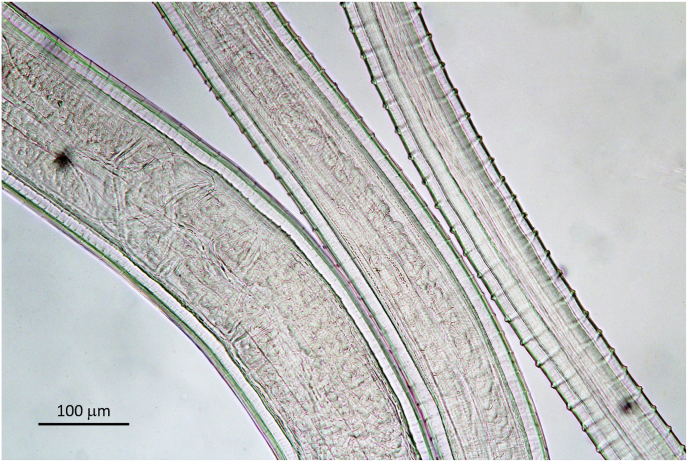
Different body width of one specimen of *Onchocerca skrjabini* in comparison. Midbody at left, tapering part in the middle, thin anterior body at right. Note Onchocerca typical structure of cuticle with ridges on the surface and striae in medulla in ratio 1 : 4.

**Fig. 12:**
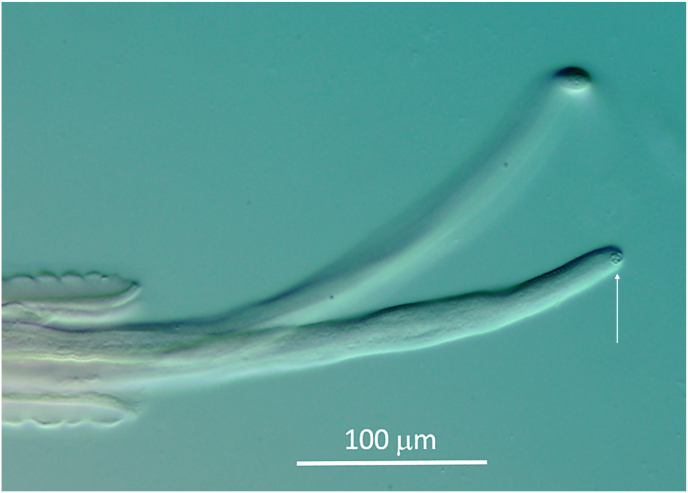
*Onchocerca skrjabini*: two microfilariae protrude from broken frontal part of a female, showing very tight setting in small anterior body part in this species. Note subapical oval mark with protruding rim on head of microfilaria.

Body length not determined, the longest single piece 180 mm long. Body sectioned in two different diameters: thin head end extending over 120 mm with diameter of 80 μm–100 μm, posteriad expanding 2.5 times to 225 μm–270 μm (Fig. 1, Fig. 11). Unpaired uterus in front end filling most of lumen of body, giving typical tight aspect in this species (Fig. 10, Fig. 12). Slight dilatation at level of nerve ring, as in male. Diameter at nerve ring 100 μm (80 μm, 100 μm). Oesophagus length 1380 μm (1500 μm, 1220 μm). The muscular part extending over 670 μm in the 1380 μm long oesophagus and 450 μm in the 1220 μm long oesophagus (Fig. 10F). Nerve ring at 300 μm (260 μm, 260 μm) from anterior end. Vulva just posterior to end of oesophagus, at 1430 μm (or just anterior, at 1300 μm, 950 μm) from anterior end. Ovejector connects to vulvar opening with a small duct (Fig. 10F).

**Fig. 13:**
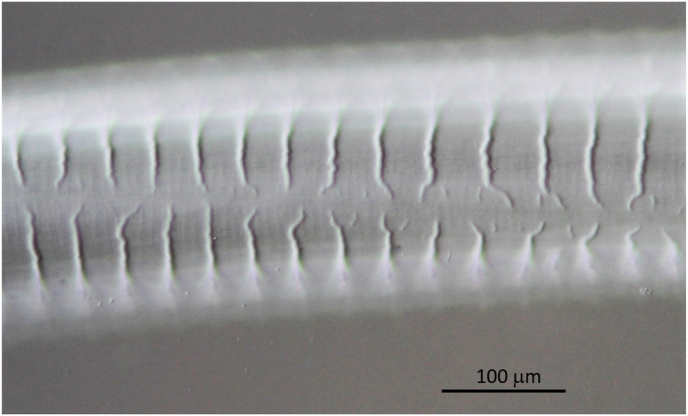
*Onchocerca skrjabini* female: cuticular ridges do not meet over the lateral line, they taper and disappear distant from each other.

Cuticle with Onchocerca-typical two layers: Outer cuticle with marked transversal annulation, which fade out over the lateral line without touching each other, without or with only rudimental bifurcations (Fig. 13). The internal medullar part of cuticle transversely striated, ratio of annular ridges: striae is mostly 1 : 4 (Fig. 10, Fig. 11). Towards head and tail end the annulation is smoothing out, becoming smaller and narrower (Fig. 10H).

Body diameter tapers rapidly towards anus. Tail with slight club-shape 170 μm (140 μm, 150 μm) in length, with diameter of 70 μm in all three specimens. Two pointed subterminal phasmids and a fine terminal knob at tail tip (Fig. 10H and I).

##### 3.1.2.3 Microfilaria

Microfilariae measured in the distal part of unpaired uterus 410–430 μm long and 10–11 μm wide. Head of microfilaria with subapical oval mark with protruding rim (Fig. 10, Fig. 12).

### 3.2 Genetic analyses

Of the 85 worms identified at the *nd*5 locus, 20 specimens were chosen for additional analysis at three mitochondrial loci and one genomic locus (Table 1).

Obtained sequence lengths and their identity with *Onchocerca* spp. from the GenBank are given in Table 3. For all five loci of *O. flexuosa* (n = 6), high sequence identities with corresponding entries in GenBank were observed. *Onchocerca jakutensis* (n = 5) could be identified at four of the five loci. A novel sequence for this species was obtained for the 5S IGS (highest identity of 93.1% with sequence of *O. gutturosa*). Isolates OS_A to OS_I (n = 9) had high identities with sequences of *O. skrjabini* from Japanese sika deer (AM779804, AM749271) at two loci (12S rDNA: 97.1–97.7, *cox*1: 97.6–98.1%). No corresponding sequences were available at the other loci, highest identities were 92–93.1% (*nd*5), 95.5–95.7% (16S rDNA) and 81.8% (5S IGS) with sequences of different Onchocerca species. At the *nd*5 locus, there was an additional high identity (98.6–98.9%) with a sequence derived from uncharacterized microfilariae (KU886068) from red deer shot in the same region (Bosch et al., 2016). Morphologically, fragments of these specimens were identified as *O. skrjabini* after Bain and Schulz-Key (1974) (7 specimens) or, following Demiaszkiewicz (1989), as *O. garmsi* (2 specimens).

The 5S IGS PCR produced amplicons of around 450 bp, but sequencing often failed, and the obtained sequences of around 150–370 bp harboured multiple ambiguous bases indicating intra-individual polymorphism. For comparison with corresponding GenBank entries, a sequence of about 160 bp length was selected (Krueger et al., 2007). A single unambiguous sequence was obtained for each the *O. skrjabini* and *O. jakutensis* specimens, and two sequences for *O. flexuosa* (Table 1, ON854655–ON854658). The *O. skrjabini* sequence harbours a 13 bp deletion which is unique compared to the other *Onchocerca* spp. sequences.

The sequences of *nd*5 and *cox*1 were translated into their corresponding protein sequences. The *cox*1 protein sequences were all identical between the three species. The *nd*5 protein sequences showed inter-species variation (13/133 amino acids). They were conserved within the species, showing no variation among the *O. jakutensis* sequences. One polymorphism each was evident among the sequences of *O. flexuosa* (V to I) and *O. skrjabini* (L to F), in both cases affecting amino acids belonging to groups of strongly similar properties.

For alignments, sequences were trimmed to lengths of corresponding GenBank entries, exemptions being indels.

**Fig. 14:**
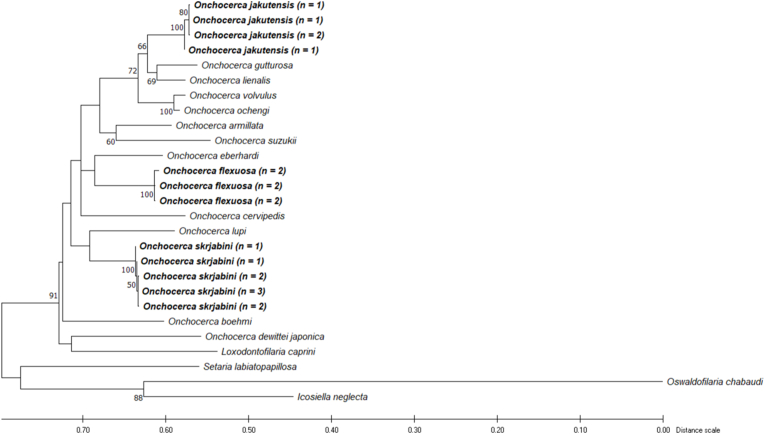
Dendrogram of Onchocerca from this study, additional Onchocerca species and *Setaria labiatopapillosa*, *Oswaldofilaria chabaudi* and *Icosiella neglecta* as outgroups based on a dataset of 935 positions of the concatenated mitochondrial *cox*1 and 12S rDNA gene, estimated by using the Maximum Likelihood method and GTR + G + I substitution model (Nei and Kumar, 2000). The tree with the highest log likelihood (−4758.48) is shown. Bootstrap values over 50 are shown next to the branches. Sequences newly generated in this study are in bold.

A phylogenetic analysis of the concatenated sequences of the partial *cox*1 gene and 12S rDNA, including eleven other *Onchocerca* spp., placed *O. jakutensis* in a cluster with *O. gutturosa* and *O. lienalis*, a clade also including *O. volvulus* and *O. ochengi* (Fig. 14). This placement of *O. jakutensis* close to *O. volvulus* is supported by analyses, with fewer available sequences, of the concatenated partial *nd*5 gene, *cox*1 gene, 12S rDNA and 16S rDNA (supp. Fig. 1) and of the 5S IGS (supp. Fig. 2).

**Fig. 15:**
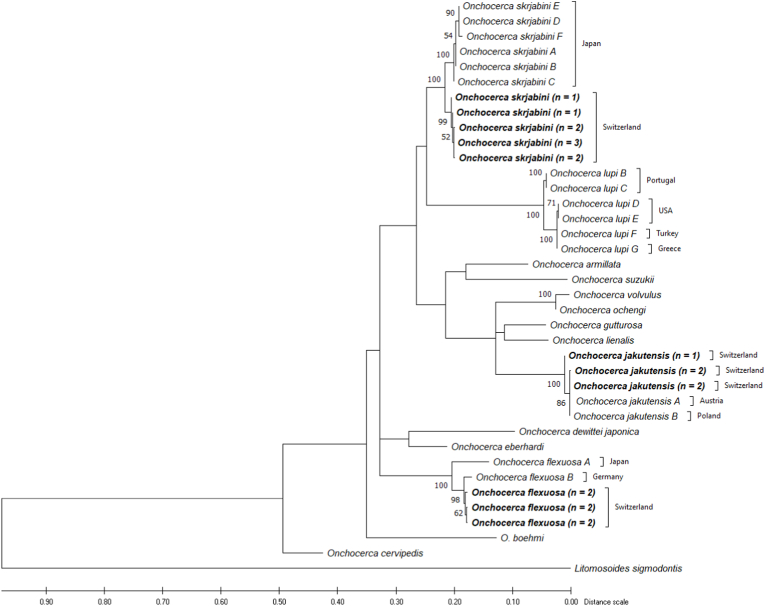
Dendrogram of Onchocerca from this study, various other Onchocerca sequences and *Litomosoides sigmodontis* as an outgroup based on a dataset of 579 positions of the mitochondrial *cox*1 gene, estimated by using the Maximum Likelihood method and TN93 + G + I substitution model (Tamura and Nei, 1993). The tree with the highest log likelihood (−2902.13) is shown. Bootstrap values over 50 are shown next to the branches. Sequences newly generated in this study are in bold.

Sequence variability at the *cox*1 (Fig. 15) and 12S rDNA loci (supp. Fig. 3) of Onchocerca specimens from different countries was obvious in *O. skrjabini* from Japan and Switzerland as well as in *O. flexuosa* originating from Japan and several European countries.

## 4 Discussion

### 4.1 Morphology

All specimens of *O. flexuosa* are well characterised and contrasted from the other *Onchocerca* spp. from the same host, considering length of oesophagus, location of vulva, form of male tail with spicule length and form, and distribution of the papillae. The morphological data given by Bain and Schulz-Key (1974) are confirmed with the description in this work. Additional typical characteristics found in this investigation are the spacious body of the female, the conspicuously swollen ovejector, the various forms of the heteromorphous cuticle and the brown colouration of aging specimens. Further, not mentioned by these authors is the curly form of the body of females (Fig. 6A). This form can also be considered as typical for *O. flexuosa*. The close up reveals stressed uteri with undulating wide integument around (Fig. 9). Schulz-Key (1975a) considered this aspect an artefact from stretching during preparation. In the present survey, stretched uteri were found regularly after enzymatic digestion of the nodules without prior handling the specimen. This was neither observed in *O. skrjabini* nor *O. jakutensis* after using the same digestion method (Fig. 6B). Schulz-Key (1975a) described the growth and decay of nodules in the host over time and the morphological changes of the females living inside. He noted that the female bodies change to the curly form in connection with the swelling of the cuticula. This might result in the barely visible or even lacking cuticular annulation (= ridges) in females noted above, also mentioned by Bain and Schulz-Key (1974) and Hidalgo et al. (2015). The illustrations given of the very different aspects of the heteromorphous cuticle in the female can be helpful in identifying pieces of worms removed from typical or atypical hosts. The aging process is accompanied by an increasing brown staining of the internal organs without involving the cuticle and terminated by multiple breakages of the body, until only crumbs of females are found. No calcification inside the parasite was seen, in contrast to *O. jakutensis* (Bosch et al., 2016).

The morphology of the *O. skrjabini* specimens investigated include most of the original description of Bain and Schulz-Key (1974) and the key in Bain (1981). However, one remarkable characteristic of this species not explicitly mentioned before is the very thin and long anterior part of the female body, which extends over at least 120 mm as measured in one specimen (Fig. 1B, arrows). The diameter increases posteriad steadily to 250–270 μm, to more than double the anterior size (Fig. 11). The unpaired uterus inside the thin anterior part fills the available space, which provides a very typical aspect of tightness for *O. skrjabini* females (Fig. 10, Fig. 12). Another species affecting cervids, *O. eberhardi*, was also reported with a narrow anterior body (Uni et al., 2007), though of only 3 mm in length.

Bain and Schulz-Key (1974) and Bain (1981) described an unclear differentiation between the muscular and the glandular part of the oesophagus. However, the present work revealed one male and two female specimens with a clear differentiation, marked by an increase of diameter by 25% and by a massive presence of glandular cells. A clearly differentiated glandular oesophagus was also depicted by Yagi et al. (1994) for females of *O. skrjabini* retrieved from dwarf goats (*Capra hircus*) from Japan. They determined this as a slight morphological difference between Japanese and European specimens. It is interesting that such differentiated individuals were also found in deer from Switzerland.

The very long and thick microfilariae distinguish *O. skrjabini* from the other three *Onchocerca* spp. in the same host. Adults of *O. skrjabini* are easily distinguishable from *O. flexuosa* as described above. The differences to *O. jakutensis* and especially to *O. garmsi* are smaller. All these three *Onchocerca* spp. have a distance between head-end to vulva of more than four times the distance from head-end to nerve ring. *Onchocerca skrjabini* differs by its much longer oesophagus (>1000 μm) from *O. jakutensis.*

The differences of *O. skrjabini* to *O. garmsi* were described, but based on the fragments of one female only available at the time (Bain and Schulz-Key, 1976). Following these authors, the female of *O. skrjabini* is slightly smaller at midbody, but the measurements overlap with those of *O. garmsi*. *Onchocerca skrjabini* has a slightly smaller oesophagus and longer microfilariae. *Onchocerca garmsi* was described having a conical tail in the female, which distinguishes it from *O. skrjabini* and *O. jakutensis*. However, Demiaszkiewicz (1989) also found a fragment of a female with a cylindrical tail in his material from sternal skin and added it to his redescription of *O. garmsi*. Demiaszkiewicz (1989) gave the first description of the male of *O. garmsi*. The characteristic precloacal papillae of the male tail depicted allows to differentiate it from *O. skrjabini*. In contrast, he also found a fragment with a male-tail without this difference to *O. skrjabini* in the sternal skin and added it to the description of *O. garmsi.* The inclusion of these additional fragments obscures the differentiation between *O. garmsi* and *O. skrjabini*, especially when only fragments are available for identification.

Following the literature, the species *O. garmsi* could be found in the sternal skin (Bain and Schulz-Key, 1976; Demiaszkiewicz, 1989). Considering the high worm burden found at this site (Fig. 2) and the here described occurrence of *O. skrjabini* in sternal skin raises the question whether the material of *O. garmsi* described by (Demiaszkiewicz, 1989) possibly was heterogenous.

In our investigation of the sternal skin, all fragments containing characteristics with taxonomic value (e.g. microfilariae, head parts, male tails) were identified as *O. skrjabini* after Bain and Schulz-Key (1974), confirmed by genetic analysis. The specimens of *O. skrjabini* in this investigation originate from the carpal skin and, in two hosts, also from the skin over the sternum. This is a new site reported for *O. skrjabini* in the deer.

### 4.2 Genetic identification

Genetic identifications at the *nd*5 locus were in good agreement with the morphological ones. A fragment of a female tail, found free in the digested sternal skin (OJ_E, Table 1), could not be identified morphologically (lack of characteristic features) and was genetically identified as *O. jakutensis*. Thus, besides the described *O. garmsi* and *O. skrjabini*, a third species can be found in the sternal skin. A further remarkable finding is the free-living female of *O. jakutensis* found in the sternal region; this is a new presentation for this species.

One male worm in this study was genetically assigned to *O. flexuosa* in a deer from which otherwise morphologically only *O. jakutensis* was identified, and no nodules were found that matched *O. flexuosa* (animal D, Table 2).

There are very few studies describing the occurrence of *O. garmsi*, and no reference sequences are deposited in GenBank. The availability of DNA sequences for clear identification would be an asset. Thus, we requested a piece of stored *O. garmsi* specimens from the two European institutions that have published on this species (W. Stefański Institute of Parasitology, Warsaw, Poland; Muséum National d’Histoire Naturelle (MNHN), Paris, France), but unfortunately both had to decline our request, because the specimens were either no longer available or present the species holotype. Thus, the morphological description of *O. garmsi* correlated with genetic data awaits further investigation.

### 4.3 Genetic analyses

Genotyping provides a relatively simple way to determine the species of a located worm specimen. Initially, only the *nd*5 gene was analysed in this study. While *O. flexuosa* and *O. jakutensis* could both be accounted for with high conformity, the presumed *O. skrjabini* sequences had a high identity with the sequence of an undetermined species, but no significant match (max. 93%) with other entries in GenBank. Therefore, the analysis was extended to a number of genetic loci used previously for species identification and phylogenetic analysis of Onchocerca (Casiraghi et al., 2001; Krueger et al., 2007).

The aim of this study was not to provide a full-scale phylogenetic analysis but to provide a basic understanding on the relationship between the species *O. flexuosa*, *O. jakutensis* and *O. skrjabini* from this study. Lefoulon et al. (2017) used the concatenated sequences of seven loci (12S rDNA, *cox*1 and five others not addressed in the present study) from thirteen *Onchocerca* spp., including four of the seven known cervid Onchocerca, to derive a phylogenetic tree. They described three clades, with the cervid *Onchocerca* spp. belonging to clade I (*O. cervipedis*) or clade II (*O. eberhardi*, *O. flexuosa, O. skrjabini*). *Onchocerca jakutensis* was not included in that analysis. Our work places it in clade three, as the only species with a cervid host. Thus, the conclusion of Lefoulon et al. (2017) that a host switch among Bovidae, Canidae and humans occurred in this clade needs reconsideration.

While *O. skrjabini* have been described in European red deer, sequences found in GenBank all stem from specimens obtained in Japan from sika deer (*Cervus nippon*) or Japanese serow (*Capricornis crispus*) (Ferri et al., 2009; Lefoulon et al., 2015) and only from two mitochondrial genes (12S rDNA, *cox*1). A phylogenetic analysis including the sequences from our study revealed two subpopulations, one from European red deer and one from Japanese serow and sika deer (Fig. 15, supp. Fig. 3). Lefoulon et al. (2017) showed the mean intraspecific nucleotide distance of the *cox*1 gene in most of the studied species to be lower than 2%. The difference between the *O. skrjabini* from Switzerland and those from Japan, with 2.48%, is slightly higher. Slight morphological differences of these Japanese isolates as compared to *O. skrjabini* from European red deer (Bain and Schulz-Key, 1974) were also described (Yagi et al., 1994), raising the question on their taxonomic status.

Genetic variability between *O. flexuosa* from two continents (Europe, Asia) at the *cox*1 locus exists (Fig. 15) but only a single sequence is available from Asia (Japan) (Abd-Ellatieff et al., 2022). 12S rDNA sequences only exist from European *O. flexuosa* isolates, without a geographic clustering.

For *O. jakutensis*, sequences and descriptions exist only from European red deer, and no zoogeographic variability is obvious.

A few technical problems appeared during genotyping. The identity of the ninth worm morphologically described in this study in detail, morphologically *O. skrjabini*, was confirmed by sequencing but the quality of these sequences was low, and they were therefore not considered for extensive genetic analysis. The 16S rDNA locus was difficult to amplify with the *O. skrjabini* specimens, and the amount of DNA and number of replication cycles had to be increased to obtain at least faint bands in gel electrophoresis. 12S rDNA sequences of *O. skrjabini* generated with the forward primer were very short or of low quality. The reverse primer worked better, although the obtained sequences were shorter (median 372 bp) than those of the other two species (median 435 bp). The reason for this might be a poly T stretch in *O. skrjabini* around 90 bp after the binding site of the forward primer, possibly leading to polymerase slippage during sequencing. Ferri et al. (2009), the authors of the *O. skrjabini* sequences in GenBank, do not mention any problems with sequencing.

Gel electrophoresis of the 5S IGS PCRs showed multiple bands. Corresponding sequences have previously been generated by cloning the PCR products (Xie et al., 1994) or by excising the band of the expected size (Masatani et al., 2021) as also done in this study. Still, sequencing was unsatisfactory. Although both forward and reverse primers were applied, consensus sequences varied considerably in length, with copious ambiguous bases, and the amplification did not work at all in some cases. Showing intra-individual polymorphism, as noted by Krueger et al. (2007), sequencing with excised bands seems not to be an appropriate method.

### 4.4 Prevalence and vectors

With the selection procedure of deer in this study, no conclusions relating the prevalence of Onchocerca in the red deer population in this region is possible. The prevalence of *O. flexuosa* in Europe varies from 30.9% in Denmark (Nielsen et al., 2022), to 96.3% in Germany, where other *Onchocerca* spp. where also found with prevalences of 82% (*O. skrjabini*), 22.9% (*O. jakutensis*) and 2% (*O. garmsi*) (Schulz-Key, 1975b). In Switzerland, the prevalence of *O. jakutensis* was found to be comparable, with 24% (Bosch et al., 2016).

Two taxa, *Simuliidae* (black flies) and *Ceratopogonidae* (biting midges, genus Culicoides), are the incriminated vectors of Onchocerca (Post et al., 2007; Wang et al., 2020). Upon ingestion by the vector, microfilaria move to the insect thorax which can be a very rapid step as shown with *O. cervicalis* in its Culicoides vector (15 min; Mellor, 1975). There, they develop into infectious L3 which move to the insect mouth parts and are deposited on the skin during the insect feeding on the vertebrate host. Morphologic discrimination of the larval stages between species is difficult. There are only few studies on genetic identification of Onchocerca other than *O. volvulus* in their natural vectors. For example, PCR primers specific for *O. fasciata* were designed to identify *C. puncticollis* as vector (Wang et al., 2020). For the three *Onchocerca* spp. infecting red deer from this study, only studies based on morphology exist. Two species of *Simuliidae* were demonstrated to harbour L3 of *O. skrjabini* (Schulz-Key and Wenk, 1981) and *O. flexuosa* (Frank et al., 1968). A re-examination of the material of the latter study also revealed the presence of *O. skrjabini* (Schulz-Key and Wenk, 1981) which was not known at the time of the study. For *Ceratopogonidae*, the data on vector competence for the three *Onchocerca* spp. are scant. After feeding on a deer naturally infected with the three species, microfilaria of *O. flexuosa* and *O. jakutensis* were identified in the thorax, but developing larvae were only identified in one insect, in which case the *Onchocerca* sp. could not be determined, and no infective L3 were detected (Schulz-Key and Wenk, 1981). Further investigations with genetic identification of L3 recovered from biting midges are needed to clarify their vector role.

Several of the examined deer showed double or triple infections with *Onchocerca* spp. Bosch et al. (2016), examining deer from the same region as in this study, found only *O. jakutensis*. They only examined the deer skins and thus possibly missed both *O. flexuosa* which remains on the carcass after skinning, and *O. skrjabini* that does not form nodules.

Taken together, the present work, in addition to Bosch et al. (2016), provides an advanced overview of the *Onchocerca* spp. found in red deer in the Grisons region in Switzerland, as morphological redescriptions and correlated multilocus genotyping.

## Declaration of competing interest

The authors declare that there is no conflict of interest.

## Figures and Tables

**figs1:**
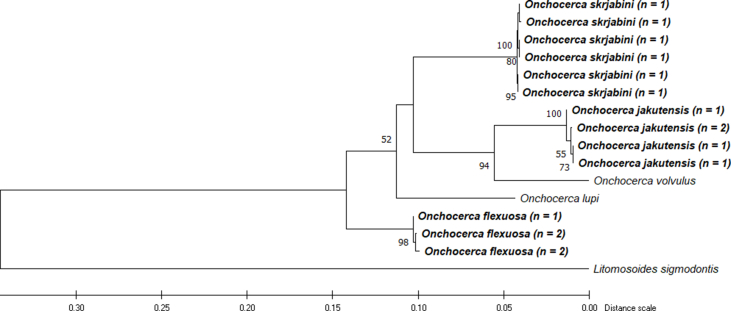
Dendrogram of Onchocerca from this study, and *O. volvulus*, *O. lupi* and *Litomosoides sigmodontis* as an outgroup based on a dataset of 1754 positions of the concatenated mitochondrial *nd*5, 12S rDNA, 16SrDNA and *cox*1 gene, estimated by using the Maximum Likelihood method and TN93 + G substitution model (Tamura and Nei, 1993). The tree with the highest log likelihood (–4959.48) is shown. T Bootstrap values over 50 are shown next to the branches. Sequences newly generated in this study are in bold.

**figs2:**
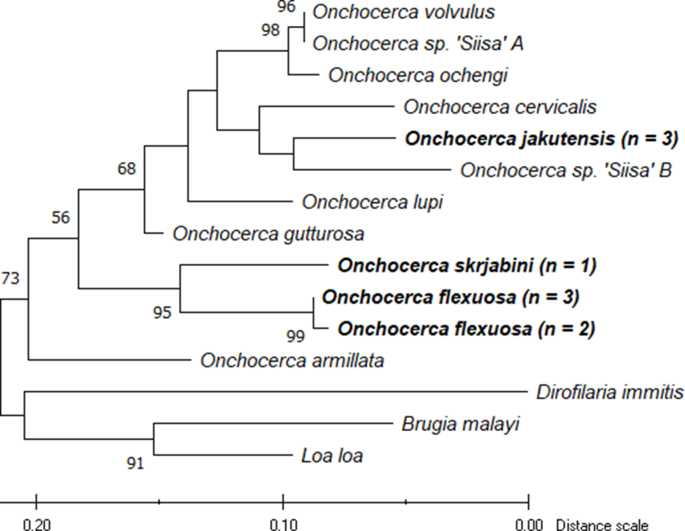
Dendrogram of Onchocerca from this study, additional Onchocerca sequences from Krueger et al. (2007), *Brugia malayi*, *Dirofilaria immitis* and *Loa loa* as outgroups based on a dataset of 163 positions of the nuclear 5S intergenic spacer sequence, estimated by using the Maximum Likelihood method and HKY + I substitution model (Hasegawa et al., 1985) similar to Krueger et al. (2007). The tree with the highest log likelihood (–890.93) is shown. Bootstrap values over 50 are shown next to the branches. Sequences newly generated in this study are in bold.

**figs3:**
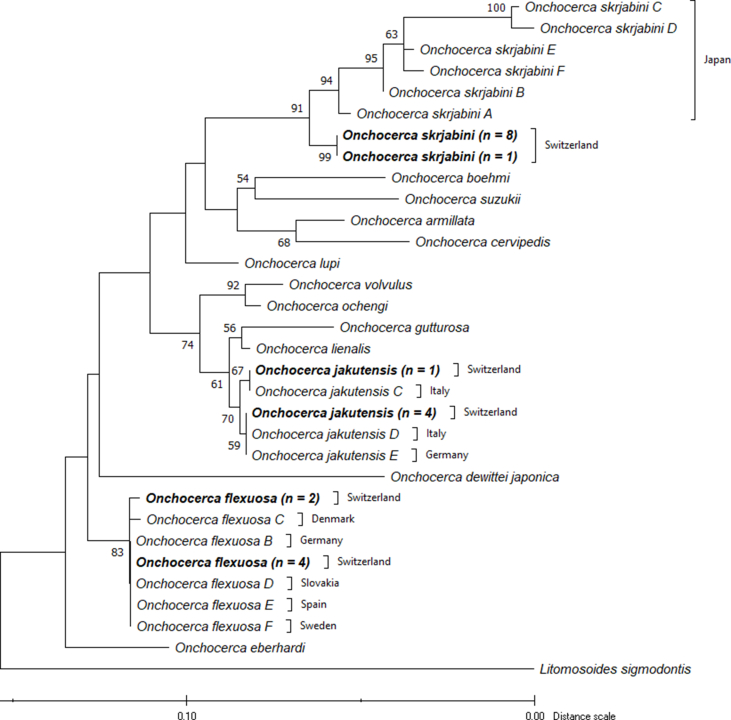
Dendrogram of Onchocerca from this study, various other Onchocerca sequences and *Litomosoides sigmodontis* as an outgroup based on a dataset of 352 positions of the mitochondrial 12S rDNA gene, estimated by using the Maximum Likelihood method and HKY + G substitution model (Hasegawa et al., 1985). The tree with the highest log likelihood (–1318.33) is shown. Bootstrap values over 50 are shown next to the branches. Sequences newly generated in this study are in bold.
