# Supplementary material for: Morphological description and multilocus genotyping of Onchocerca spp. in red deer (Cervus elaphus) in Switzerland
Source: Int J Parasitol Parasites Wildl. 2022 Nov 3;19:273–84. doi: 10.1016/j.ijppaw.2022.10.004 (PMC9676152; doi:10.1016/j.ijppaw.2022.10.004)
Supplement: Multimedia component 1 [file mmc1.docx]

Supp. Table 1: PCR-conditions used

- Hot start 95 °C 15 min
- Denaturation 94 °C 30 sesc
- Annealing AT 90 sec 35x
- Extension 72 °C 60 sec
- Extension 72 °C 10 min
- Cooling 4 °C ∞

| Gene | Primer name | Direction | Sequence (5’ – 3’) | Reference | Annealing temperature |
| --- | --- | --- | --- | --- | --- |
| ND5 | ND5OvA | Forward | TTGGTTGCCTAAGGCTATGG | Morales-Hojas et al. (2006) | 58 °C |
|  | ND5OvC | Reverse | CCCCTAGTAAACAACAAACCACA |  |  |
| 12S rDNA | 12SovC | Forward | TCGGCTATGCGTTTTAATTTT | Morales-Hojas et al. (2006) | 54 °C |
|  | 12SovB | Reverse | CAACTTACGCCCCTTTAGGC |  |  |
| 16S rDNA | 16SovC | Forward | AGCCTTAGCGTGATGGCATA | Morales-Hojas et al. (2006) | 54 °C |
|  | 16SovB | Reverse | ACCCACATTGCATTCCTTTC |  |  |
| COX1 | COIintF | Forward | TGATTGGTGGTTTTGGTAA | Casiraghi et al. (2001) | 52 °C |
|  | COIintR | Reverse | ATAAGTACGAGTATCAATATC |  |  |
| 5S IGS | S2 | Forward | GTTAAGCAACGTTGGGCCTGG | Xie et al. (1994) | 57 °C |
|  | S16 | Reverse | TTGACAGATCGGACGAGATG |  |  |

Supplementary Table 2: GenBank accession numbers of additional sequences of filarial species used in the study

| **Filarial species** | **Host species** | **Country of origin** | **Locus** | | | | |
| --- | --- | --- | --- | --- | --- | --- | --- |
|  |  |  | 12S rDNA | COX1 | ND5 | 16S rDNA | 5S IGS |
| *Brugia malayi* | *Meriones unguiculatus* | USA, Labstrain originating from Malaysia |  |  |  |  | L36060 |
| *Dirofilaria immitis* | Unknown | USA |  |  |  |  | U31637 |
| *Icosiella neglecta* | *Pelophylax ridibunda* | Ukraine | KP760333 | KP760188 |  |  |  |
| *Litomosoides sigmodontis* | Unknown | Labstrain originating from Cameroon | AP017689 | AP017689 | AP017689 | AP017689 |  |
| *Loa loa* | Unknown | USA |  |  |  |  | U31638 |
| *Loxodontofilaria caprini* | *Capricornis crispus* | Japan | AM779817 | AM749237 |  |  |  |
| *Onchocerca armillata* | *Bos taurus* | Cameroon | KP760200 | KP760345 |  |  |  |
| *Onchocerca armillata* | *Bos taurus* | Cameroon |  |  |  |  | DQ523782 |
| *Onchocerca boehmi* | *Equus caballus* | Italy | KX853323 | KX853315 |  |  |  |
| *Onchocerca cervicalis* | *Equus caballus* | Unknown |  |  |  |  | U13678 |
| *Onchocerca cervipedis* | *Alces americanus* | Canada | KX853324 | KX853316 |  |  |  |
| *Onchocerca dewittei japonica* | *Sus scrofa leucomystax* | Japan | KP760203 | KP760349 |  |  |  |
| *Onchocerca eberhardi* | *Cervus nippon* | Japan | AM749268 | KP760346 |  |  |  |
| *Onchocerca flexuosa* A | *Capricornis crispus/ Cervus nippon** | Japan |  | LC318284 |  |  |  |
| *Onchocerca flexuosa* B | *Cervus elaphus* | Germany | HQ214004 | HQ214004 |  |  |  |
| *Onchocerca flexuosa* C | *Cervus elaphus* | Denmark | MZ714154 |  |  |  |  |
| *Onchocerca flexuosa* D | *Cervus elaphus* | Slovakia | MK391035 |  |  |  |  |
| *Onchocerca flexuosa* E | *Cervus elaphus* | Spain | JQ733522 |  |  |  |  |
| *Onchocerca flexuosa* F | *Cervus elaphus* | Sweden | LT732683 |  |  |  |  |
| *Onchocerca gutturosa* | *Bos taurus* | Cameroon | KP760201 | KP760347 |  |  |  |
| *Onchocerca gutturosa* | *Bos taurus* | Uganda |  |  |  |  | DQ523785 |
| *Onchocerca jakutensis* A | *Cervus elaphus* | Austria |  | KT001213 |  |  |  |
| *Onchocerca jakutensis* B | *Homo sapiens* | Poland |  | MK491767 |  |  |  |
| *Onchocerca jakutensis* C | *Cervus elaphus* | Italy | HQ717720 |  |  |  |  |
| *Onchocerca jakutensis* D | *Cervus elaphus* | Italy | HQ717719 |  |  |  |  |
| *Onchocerca jakutensis* E | *Cervus elaphus* | Germany | DQ523745 |  |  |  |  |
| *Onchocerca lienalis* | *Bos taurus* | Wales | KX853325 | KX853317 |  |  |  |
| *Onchocerca lupi* A | *Canis lupus familiaris* | USA | NC_056960 | NC_056960 | NC_056960 | NC_056960 |  |
| *Onchocerca lupi* B | *Canis lupus familiaris* | Portugal |  | KC686701 |  |  |  |
| *Onchocerca lupi* C | *Canis lupus familiaris* | Portugal |  | KX853327 |  |  |  |
| *Onchocerca lupi* D | *Canis lupus familiaris* | USA |  | KX853330 |  |  |  |
| *Onchocerca lupi* E | *Canis lupus familiaris* | USA |  | KP283477 |  |  |  |
| *Onchocerca lupi* F | *Homo sapiens* | Turkey |  | HQ207644 |  |  |  |
| *Onchocerca lupi* G | *Canis lupus familiaris* | Greece |  | KC686702 |  |  |  |
| *Onchocerca lupi H* | *Canis lupus familiaris* | Hungary |  |  |  |  | AJ311527 |
| *Onchocerca ochengi* | *Bos taurus* | Cameroon | KP760202 | KP760348 |  |  |  |
| *Onchocerca ochengi* | *Bos taurus* | Cameroon |  |  |  |  | DQ523781 |
| *Onchocerca skrjabini* A | *Cervus nippon* | Japan | AM779804 | AM749269 |  |  |  |
| *Onchocerca skrjabini* B | *Cervus nippon* | Japan | AM779805 | AM749271 |  |  |  |
| *Onchocerca skrjabini* C | *Cervus nippon* | Japan | AM779806 | AM749270 |  |  |  |
| *Onchocerca skrjabini* D | *Capricornis crispus* | Japan | AM779807 | AM749273 |  |  |  |
| *Onchocerca skrjabini* E | *Capricornis crispus* | Japan | AM779808 | AM749272 |  |  |  |
| *Onchocerca skrjabini* F | *Capricornis crispus* | Japan | AM779809 | AM749274 |  |  |  |
| *Onchocerca sp. 'Siisa'* A | *Simulium damnosum s.l.* | Uganda |  |  |  |  | DQ523780 |
| *Onchocerca sp. 'Siisa'* B | *Simulium damnosum s.l.* | Uganda |  |  |  |  | DQ523778 |
| *Onchocerca suzukii* | *Capricornis crispus* | Japan | KX853333 | KX853315 |  |  |  |
| *Onchocerca volvulus* | *Homo sapiens* | West Africa | NC_001861 | NC_001861 | NC_001861 | NC_001861 |  |
| *Onchocerca volvulus* | *Homo sapiens* | Uganda |  |  |  |  | AF325546 |
| *Oswaldofilaria chabaudi* | *Tropiduris torquatus* | Brasil | KP760350 | KP760204 |  |  |  |
| *Setaria labiatopapillosa* | *Bos taurus* | Cameroon | KP760354 | KP760208 |  |  |  |

*discrepancy between GenBank entry and corresponding paper

Supp. Table 3: Composition of nodules and worms analysed

| ***Deer*** | ***Nodule*** | | ***Nr. of females (Nr. sequenced)*** | | ***Sequence result*** | | ***Nr. of males***  ***(Nr. sequenced)*** | | ***Sequence result*** | |
| --- | --- | --- | --- | --- | --- | --- | --- | --- | --- | --- |
| A | 1 | | 1 (1) | | *O. jakutensis* | | 1 (0) | |  | |
|  | 2 | | 1 (1) | | *O. jakutensis* | | 0 | |  | |
|  | 3 | | 1 (1) = OJ_A | | *O. jakutensis* | | 0 | |  | |
|  | 4 | | 1 (1) | | *O. jakutensis* | | 2 (0) | |  | |
|  | 5 | | 1 (1) | | *O. jakutensis* | | 0 | |  | |
|  | 6 | | 1 (1) | | *O. jakutensis* | | 0 | |  | |
|  | 7 | | 1 (1) | | *O. jakutensis* | | 1 (0) | |  | |
| **Total** | **7** | | **7 (7)** | |  | | **4 (0)** | |  | |
| B | 1 | | 1 (1) | | *O. flexuosa* | | 0 | |  | |
|  | 2 | | 1 (1) | | *O. flexuosa* | | 0 | |  | |
|  | 3 | | 1 (1) = OF_B | | *O. flexuosa* | | 1 (1) | | *O. flexuosa* | |
|  | 4 | | 0 | |  | | 2 (1) | | *O. flexuosa* | |
|  | 5 | | 1 (1) | | *O. flexuosa* | | 0 | |  | |
|  | 6 | | 1 (1) | | *O. flexuosa* | | 1 (1) | | *O. flexuosa* | |
|  | 7 | | 1 (1) | | *O. flexuosa* | | 1 (1) | | *O. flexuosa* | |
|  | 8 | | 0 | |  | | 2 (1) | | *O. flexuosa* | |
|  | 9 | | 1 (1) = OF_A | | *O. flexuosa* | | 0 | |  | |
|  | 10 | | 1 (1) | | *O. flexuosa* | | 0 | |  | |
|  | 11 | | 1 (1) | | *O. flexuosa* | | 0 | |  | |
|  | 12 | | 1 (1) | | *O. flexuosa* | | 1 (1) | | *O. flexuosa* | |
|  | 13 | | 1 (1) | | *O. flexuosa* | | 1 (1) | | *O. flexuosa* | |
|  | 14 | | 1 (1) | | *O. flexuosa* | | 2 (1) | | *O. flexuosa* | |
|  | 15 | | 1 (1) | | *O. flexuosa* | | 1 (1) | | *O. flexuosa* | |
|  | 16 | | n. i. | |  | | 1 (1) = OF_E | | *O. flexuosa* | |
| **Total** | **16** | | **13 (13)** | |  | | **13 (10)** | |  | |
| C |  | | 2 (1) = OJ_B | | *O. jakutensis* | | 5 (4) | | *O. jakutensis* | |
| **Total** | **1** | | **2 (1)** | |  | | **5 (4)** | |  | |
| D | 1 | | 1 (1) | | *O. jakutensis* | | 0 | |  | |
|  | 2 | | 1 (1) | | *O. jakutensis* | | 2 (2) | | *O. jakutensis* | |
|  | 3 | | 1 (1) | | *O. jakutensis* | | 1 (1) | | *O. flexuosa* | |
|  | 4 | | 1 (1) | | *O. jakutensis* | | 0 | |  | |
|  | 5 | | 1 (1) | | *O. jakutensis* | | 0 | |  | |
|  | 6 | | 1 (1) | | *O. jakutensis* | | 0 | |  | |
|  | 7 | | 1 (1) | | *O. jakutensis* | | 0 | |  | |
|  | 8 | | 1 (1) = OJ_C | | *O. jakutensis* | | 0 | |  | |
|  | 9 | | 1 (1) | | *O. jakutensis* | | 0 | |  | |
|  | 10 | | 1 (1) | | *O. jakutensis* | | 0 | |  | |
|  | 11 | | 1 (1) | | *O. jakutensis* | | 0 | |  | |
|  | Free | | 1 (1) = OJ_E | | *O. jakutensis* | |  | |  | |
|  | Free | |  | |  | | 1 (1) = OS_A | | *O. skrjabini* | |
|  | Free | | 1 (1) = OS_B | | *O. skrjabini* | |  | |  | |
|  | Free | | 1 (1) = OS_H | | *O. skrjabini* | |  | |  | |
| **Total** | **11** | | **13 (13)** | |  | | **4 (4)** | |  | |
| E | | 1 | | 1 (1) | | *O. jakutensis* | | 2 (1) | | *O. jakutensis* |
|  |  | 2 | | 1 (1) | | *O. flexuosa* | | 0 | |  |
|  |  | 3 | | 1 (1) | | *O. jakutensis* | | 2 (1) | | *O. jakutensis* |
|  |  | 4 | | 1 (1) | | *O. jakutensis* | | 0 | |  |
|  |  | 5 | | 0 | |  | | 2 (1) | | *O. jakutensis* |
|  |  | 6 | | 1 (1) = OF_C | | *O. flexuosa* | | 0 | |  |
|  |  | 7 | | 1 (1) | | *O. flexuosa* | | 0 | |  |
|  |  | 8 | | 1 (1) | | *O. flexuosa* | | 0 | |  |
|  |  | 9 | | 1 (1) | | *O. flexuosa* | | 0 | |  |
|  |  | 10 | | 1 (1) = OF_F | | *O. flexuosa* | | n. i. | |  |
|  |  | Free | |  | |  | | 1 (1) = OS_C | | *O. skrjabini* |
|  |  | Free | | 1 (1) = OS_F | | *O. skrjabini* | |  | |  |
|  |  | Free | | 1 (1) = OS_I | | *O. skrjabini* | |  | |  |
| **Total** | | **10** | | **11 (11)** | |  | | **7 (4)** | |  |
| F | | 1 | | 1 (1) = OJ_D | | *O. jakutensis* | | 1 (1) | | *O. jakutensis* |
|  |  | 2 | | 1 (1) | | *O. jakutensis* | | 1 (1) | | *O. jakutensis* |
|  |  | 3 | | 1 (1) | | *O. jakutensis* | | 0 | |  |
|  |  | 4 | | 1 (1) | | *O. jakutensis* | | 2 (1) | | *O. jakutensis* |
|  |  | 5 | | 1 (1) | | *O. jakutensis* | | 2 (1) | | *O. jakutensis* |
|  |  | 6 | | 1 (1) | | *O. jakutensis* | | 0 | |  |
|  |  | 7 | | 1 (1) | | *O. jakutensis* | | 0 | |  |
| **Total** | | **7** | | **7 (7)** | |  | | **6 (4)** | |  |
| G | | 1 | | 1 (1) | | *O. jakutensis* | | 1 (0) | |  |
|  |  | 2 | | 1 (1) | | *O. jakutensis* | | 1 (0) | |  |
|  |  | 3 | | n. i. | |  | | 1 (1) = OF_D | | *O. flexuosa* |
| **Total** | | **2** | | **2 (2)** | |  | | **3 (1)** | |  |
| H | | Free | | 1 (1) = OS_D | | *O. skrjabini* | |  | |  |
|  |  | Free | | 1 (1) = OS_E | | *O. skrjabini* | |  | |  |
|  |  | Free | |  | |  | | 1 (1) = OS_G | | *O. skrjabini* |
| **Total** | |  | | **2 (2)** | |  | | **1 (1)** | |  |
| **Final total** | | **55** | | **58 (57)** | |  | | **43 (28)** | |  |

n. i.: not investigated
